# Two-Hourly Repositioning for Prevention of Pressure Ulcers in the Elderly: Patient Safety or Elder Abuse?

**DOI:** 10.1007/s11673-018-9892-3

**Published:** 2019-01-22

**Authors:** Catherine A. Sharp, Jennifer S. Schulz Moore, Mary-Louise McLaws

**Affiliations:** 1 School of Public Health and Community Medicine, University of New South Wales, 3rd Floor Samuels Building, Sydney, NSW 2052 Australia; 2 Faculty of Law, University of New South Wales, Sydney, Australia

**Keywords:** Aged care, Dementia, Law, Guidelines, Restraints

## Abstract

For decades, aged care facility residents at risk of pressure ulcers (PUs) have been repositioned at two-hour intervals, twenty-four-hours-a-day, seven-days-a-week (24/7). Yet, PUs still develop. We used a cross-sectional survey of eighty randomly selected medical records of residents aged ≥ 65 years from eight Australian Residential Aged Care Facilities (RACFs) to determine the number of residents at risk of PUs, the use of two-hourly repositioning, and the presence of PUs in the last week of life. Despite 91 per cent (73/80) of residents identified as being at risk of PUs and repositioned two-hourly 24/7, 34 per cent (25/73) died with one or more PUs. Behaviours of concern were noted in 72 per cent (58/80) of residents of whom 38 per cent (22/58) were restrained. Dementia was diagnosed in 70 per cent (56/80) of residents. The prevalence of behaviours of concern displayed by residents with dementia was significantly greater than by residents without dementia (82 per cent v 50 per cent, p = 0.028). The rate of restraining residents with dementia was similar to the rate in residents without dementia. Two-hourly repositioning failed to prevent PUs in a third of at-risk residents and may breach the rights of all residents who were repositioned two-hourly. Repositioning and restraining may be unlawful. Rather than only repositioning residents two-hourly, we recommend every resident be provided with an alternating pressure air mattress.

## Introduction

Pressure ulcers (PUs), also known as decubitus ulcers, pressure sores, pressure injuries or bedsores, are “localized injuries to the skin and/or underlying tissue usually over a bony prominence, as a result of pressure, or pressure in combination with shear” (National Pressure Ulcer Advisory Panel, European Pressure Ulcer Advisory Panel, and Pan Pacific Pressure Injury Alliance 2014, 12). Pressure ulcers can start to develop deep in the body when sitting (Bliss 2004; Thorfinn, Sjoberg, and Lidman 2009; Linder-Ganz et al. 2007) or lying in one position without pressure relief (Bliss and Simini 1999; Bliss 2004; Schoonhoven, Defloor, and Grypdonck 2002; Moore and Cowman 2012). Notably, PUs may not become visible on the skin for several hours (Schoonhoven, Defloor, and Grypdonck 2002) or up to three weeks (Allman, Goode, and Patrick 1995; Sundin et al. 2000).

Pressure ulcers are a common condition at the end of life for residents in residential aged care facilities (RACFs) (Doupe et al. 2016; Jaul 2010; Jaul and Calderon-Margalit 2015). They can be extremely painful (Ahn, Stechmiller, and Horgas 2013; Pieper, Langemo, and Cuddigan 2009; Kwong et al. 2011) and the pain is often unrelenting (Woo et al. 2017; Gorecki et al. 2009; Bliss 2009). Screening residents for risk of PU as a strategy to introduce prevention interventions involves using a numerical screening tool and clinical judgement. However, the level of evidence for classifying residents using risk criteria from numerical tools is low (Sharp and McLaws 2006; Anthony et al. 2010; Chou et al. 2013). Unlike other screening tools in medicine, none of the commonly used PU risk screening tools has undergone rigorous testing for reliability or validity and none has been identified as a good predictor of PUs (Sharp and McLaws 2006; Franks, Moffatt, and Chaloner 2003; Defloor and Grypdonck 2004; Webster et al. 2011; Black 2015). Many nurses do not use screening tools (Samuriwo and Dowding 2014; Sharp et al. 2005; Wann-Hansson, Hagell, and Willman 2008; Defloor and Grypdonck 2004; Webster, Gavin, and Nicholas 2010; Sharp et al. 2000) but screen residents using clinical judgement to determine PU risk (Sharp and White 2015; Sharp and McLaws 2006; Anthony et al. 2010; Sharp et al. 2005, 2000).

One reason for the poor predictive ability of screening tools is many contain items that are not needed, do not contain items that would be clinically useful, have sub-scores that are not scaled optimally, and include items that are not independent predictors (Anthony et al. 2010; Sharp and McLaws 2006). Moreover, routine PU risk screening and screening for many other risks, such as pain in residents with dementia (Herr, Bjoro, and Decker 2006), falls (Lester et al. 2008), delirium (Weinhouse et al. 2009; O’Keeffe and Lavan 1999) and nutrition (Hamirudin et al. 2013) have become commonplace and consume considerable nursing resources with little benefit (Webster, Gavin, and Nicholas 2010). Screening tools may have some value in detecting residents who will develop PUs but have a high level of false positive responses. Falsely labelling residents as at-risk has resource implications when providing prevention strategies for residents who will not develop PUs (Franks, Moffatt, and Chaloner 2003).

The international *Prevention and Treatment of Pressure Ulcers: Clinical Practice Guideline* (henceforth *Clinical Practice Guideline*) recommends that screening is conducted “as soon as possible and within a maximum of eight hours after admission” (National Pressure Ulcer Advisory Panel, European Pressure Ulcer Advisory Panel, and Pan Pacific Pressure Injury Alliance 2014, 16). But this recommendation for time to screen is not evidence-based or physiologically-based (Black 2015; Sharp and White 2015). Inflammatory mediators are released within the first four hours of exposure to pressure (Stojadinovic et al. 2013). Therefore, the consequences of leaving a patient or resident for up to eight hours prior to screening may increase the risk of PUs because unrelieved pressure anywhere from half an hour (Bliss 1994) to six hours is sufficient for PUs to develop (Salcido 2004; Linder-Ganz and Gefen 2007; Gefen 2008; Exton-Smith, Overstall, and Wedgewood 1982).

When physical or chemical restraints of residents have been associated with PU development, it is thought to be likely correlated with the residents’ inability to move to relieve pressure (Mott, Poole, and Kenrick 2005; Brower 1993). Residents may be restrained physically by belts, vests, and jackets in an effort to protect them from inflicting injury on themselves or others (Ben Natan et al. 2010; Chaves et al. 2007; Brower 1993) and to prevent residents from falling (Bellenger et al. 2017). Highly restrictive restraints have been associated with death from asphyxiation (Chaves et al. 2007), neck compression, and entrapment (Bellenger et al. 2017). The development of PUs in restrained residents in RACFs increases the facility’s exposure to legal action (Voss et al. 2005; Toolan et al. 2014; Brower 1993; Tsokos, Heinemann, and Puschel 2000). Successful litigants in Australia (Nelson 2003), the United Kingdom (Toolan et al. 2014) and the United States (Stevenson and Studdert 2003) may be awarded compensatory and/or exemplary damages. In the United States, lawsuits against RACFs have risen and nearly half have involved wrongful death, PUs, or both (Stevenson and Studdert 2003).

Commonly, once a patient or resident has been identified as at risk of PUs, two-hourly repositioning, twenty-four-hours-a-day, seven-days-a-week (24/7) is routinely instituted as the preventive strategy in hospitals and RACFs (Hagisawa and Ferguson-Pell 2008; Krapfl and Gray 2008; Clark 1998; Rich, Margolis, and Shardell 2011; Defloor, De Bacquer, and Grypdonck 2005; Gillespie et al. 2014). However, the evidence for this strategy is equivocal (Defloor, De Bacquer, and Grypdonck 2005; Gunningberg and Stotts 2008; Versluysen 1985; Rich, Margolis, and Shardell 2011) because PUs have continued to develop (Krapfl and Gray 2008; Gillespie et al. 2014). One explanation for the low level of evidence for two-hourly repositioning is an absence of evidence for the optimal frequency for repositioning (Peterson et al. 2013; Krapfl and Gray 2008; Clark 1998; Gillespie et al. 2014; Rich, Margolis, and Shardell 2011). Yet, alternating pressure air mattresses (APAMs) have been shown to prevent PUs (Bliss, McLaren, and Exton-Smith 1967; Manzano et al. 2013; Exton-Smith, Overstall, and Wedgewood 1982). In contrast to a punitive approach (such as exemplary damages), regulatory tools can be used in the spirit of prevention. For example, section 3 (item 3.2) of the Australian *Quality of Care Principles 2014* (*Cth*) could be used to advance efforts to prevent PUs in RACFs by providing residents with “an air mattress appropriate to each care recipient’s condition.”

Although the cost to provide PU prevention to patients at risk can significantly increase health care services’ budgets, the costs to treat a severe PU were found to be substantially higher (Demarré et al. 2015). Lapsley and Vogels (1996) identified the average additional bed day cost in Australia associated with PUs in 1990–1992 at A$483. Even when patients are discharged, the additional costs continue due to outpatient and/or home visits from the community nurse (Lapsley and Vogels 1996). In one systematic review, the cost of PU prevention and treatment differed considerably between studies. More recently, the cost of PUs in both hospital and RACFs in Australia totalled US$1.65 billion, with a standard deviation of US$1.05 billion (Graves and Zheng 2014).

Twenty-three years ago, APAMs were found to be more cost-effective than repositioning (Xakellis, Frantz, and Lewis 1995), and in a very recent randomized, controlled study, APAMs were found to be more cost-effective than a foam mattress in preventing PUs in elderly patients bedridden for more than fifteen hours per day (Sauvage et al. 2017).

According to the *Clinical Practice Guideline*, “if changes in skin condition should occur, the repositioning care plan needs to be re-evaluated” (National Pressure Ulcer Advisory Panel, European Pressure Ulcer Advisory Panel, and Pan Pacific Pressure Injury Alliance 2014, 23)*.* The focus on skin condition is used as an early sign of pressure damage (Stojadinovic et al. 2013; Gefen, Farid, and Shaywitz 2013) and for residents’ skin tolerance for two-hourly routine repositioning (Wann-Hansson, Hagell, and Willman 2008). The focus on skin condition may overshadow the importance of immobility (Sharp and McLaws 2006; Tschannen et al. 2012; Woo et al. 2017; Allman, Goode, and Patrick 1995) and duration of unrelieved pressure as risk factors for PUs (Linder-Ganz et al. 2007; Lindgren et al. 2004; Baumgarten et al. 2006; Sharp and White 2015; Bouten et al. 2003; Kemp et al. 1990). Having previously examined PU prevalence and prevention practices in hospitals in a major area health service (Sharp et al. 2005, 2000) and a community nursing service in Sydney, Australia (Sharp 2006), we focused this current survey on the elderly in Sydney RACFs.

## Methods

Our aim of reviewing records of residents was to measure the magnitude of the routine practice of two-hourly repositioning and whether this successfully prevented PUs. A retrospective cross-sectional analytical survey design for data collection from medical records was used because a randomized control trial was not ethically feasible. In addition, given that recent inquiries have uncovered examples of institutional elder abuse (New South Wales Parliament 2016; Australian Law Reform Commission 2017; Waldegrave 2015; Australian Law Reform Commission 2016; Forrester and Williams 2008) we analysed the regulatory and policy landscape in Australia and New Zealand. As common law jurisdictions, these countries enable assessment of the legal and ethical implications of two-hourly repositioning and the use of restraints. Ethics approval was provided by the UNSW Australia Human Research Ethics Committee HC, number HC14163.

### Sample of Residents

Our pilot study identified that there was no difference from week to week over twelve weeks during residency for the practice of two-hourly repositioning 24/7. Residents live in RACFs for a mean number of 864 days from admission to death (range 38 to 3459 days), consequently the notes are copious. Notes include nursing entries, usually written three times a day, and entries by medical staff, physiotherapists, podiatrists, and others. Therefore, the last week of life was chosen rather than a full review of all boxes of notes. Globally, studies of PU prevalence in elders have been carried out by more than one nurse/researcher on each occasion. Numbers ranged from 95 residents in one RACF in Canada (Davis and Caseby 2001), 827 in Spain (Casimiro, Garcia-de-Lorenzo, and Usan 2002), 1100 residents in a group of RACFs in Ireland (Moore and Cowman 2012), and 3459 in Germany (Wilborn, Halfens, and Dassen 2006). These studies have produced varied results. A random selection of records was reviewed for eighty residents aged ≥ 65 years who died between April 2011 and April 2014.

One author (CAS) reviewed the records for age at death, sex, source of admission, risk screening for PU on admission to the RACF, prevalence of PU on admission to the RACF, the type of PU prevention practices during the last week of life (including two-hourly repositioning 24/7), and incidence of PU between time of admission and death. Records for the same eighty residents were examined for the last week of life for a diagnosis of dementia and carers perceptions of behaviours of concern and form of restraint: chemical, physical, or both.

#### Pressure ulcer risk

Risk of PUs were classified from the records in the last week of life, including (i) immobility, determined from records as a resident requiring assistance with walking by two staff members, bedfast or wheelchair bound, and (ii) records stating “two-hourly pressure area care (PAC).” Otherwise residents were classified not at-risk where records indicated the resident could walk unaided or with a walker.

#### Dementia

Dementia is an umbrella term used to describe a syndrome associated with more than a hundred conditions characterized by the impairment of brain functions, including language, memory, perception, personality, and cognitive skills (Australian Institute of Health and Welfare 2012, 2). Only medical practitioners’ documentation of a diagnosis of dementia in the notes was collected.

#### Behaviours of Concern

In this paper we will refer to “challenging behaviours” as “behaviours of concern.” These behaviours may include agitation (Kong 2005; Opie, Doyle, and O’Connor 2002; Cohen-Mansfield, Marx, and Rosenthal 1989) pacing (Nakaoka et al. 2010), spitting, cursing (Cohen-Mansfield, Marx, and Rosenthal 1989), verbal aggression (Lemay and Landreville 2010; Cohen-Mansfield, Marx, and Rosenthal 1989; Opie, Doyle, and O’Connor 2002), hitting, kicking, scratching, pushing, and screaming (Low, Brodaty, and Draper 2002). Residents who scream may not be suffering from poorer health than other residents in the RACF, but nursing staff perceive these people to be suffering from pain, not distress from disrupted sleep (Cohen-Mansfield, Werner, and Marx 1990). These behaviours have been included because any or all of them may be an indication that a resident is in pain (Pieper et al. 2011; Opie, Doyle, and O’Connor 2002). Records were examined for behaviours of concern.

#### Restraints

Restraints used were physical or chemical, including being tied to a chair or sedation. Only medical practitioners can order physical and chemical restraints for residents displaying behaviours of concern. Restraints are then implemented and documented by nursing staff. Records were examined for physical and/or chemical restraint.

#### Staging of Pressure Ulcers

Pressure ulcer staging according to the international *Clinical Practice Guideline* requires a visual assessment of skin (National Pressure Ulcer Advisory Panel, European Pressure Ulcer Advisory Panel, and Pan Pacific Pressure Injury Alliance 2014). However, care staff do not routinely document the stage of PUs in the notes. Therefore PUs were categorized according to nursing records as either stage 1 (intact skin) or stage 2, an open wound PU (describes stages 2, 3 and 4, PUs of different depths of tissue destruction down to bone in the international *Clinical Practice Guideline*) (National Pressure Ulcer Advisory Panel, European Pressure Ulcer Advisory Panel, and Pan Pacific Pressure Injury Alliance 2014). This level of reporting is now aligned with the benchmark set by the Australian Council on Healthcare Standards (ACHS) for Surgical Site Infection (SSI) Definition. For example, infection that involves both superficial and deep incisional sites is now classified as deep incisional SSI (Australian Commission on Safety and Quality in Health Care 2006). The descriptors provide consensus-based differentiation of PUs from other types of wounds, such as skin tears and venous ulcers (National Pressure Ulcer Advisory Panel, European Pressure Ulcer Advisory Panel, and Pan Pacific Pressure Injury Alliance 2014).

#### Statistical Analysis

Data were entered onto a data collection spreadsheet and exported to STATA SE version 14 (StataCorp 2015) for all statistical analyses. Descriptive statistics included frequency, range, means, proportions, 95 per cent Confidence Interval (95 per cent CI) around proportions, and standard deviations (SD) around means. Confidence intervals were adjusted to account for clustering by facility using Taylor linearized standard errors. Immobility as a risk factor (Sharp and McLaws 2006) was examined for prevalence and incidence of PU. Differences in the frequency of PU in residents with documentation of dementia and chemical or physical restraints were examined qualitatively using field observation to provide contextual richness. Corrected Pearson chi square, converted to an F test, was used to test for differences between groups.

### Sample of Materials Related to Elder Law

We sampled legal and policy materials such as cases, legislation, government reports, guidelines, regulations, and principles that were relevant to aged care and elder abuse. These materials broadly fall into an area of law called “elder law.” We also included secondary source commentary on the legal and policy documents that we subsequently identified. We reviewed these documents to assess the legal implications of two-hourly repositioning and the use of restraints.

## Definitions of Abuse

Institutional abuse of the elderly is a widely recognized type of elder abuse (McDonald et al. 2012; Mosqueda, Heath, and Burnight 2001). However, there is no authoritative definition of institutional elder abuse. Typically, institutional abuse is described as abuse or maltreatment of a person by or from a system of power (Mosqueda, Heath, and Burnight 2001). Such abuse may be physical, psychological, financial, and/or sexual. “Elder abuse” is also the terminology used by the Australian Law Reform Commission (ALRC) (Australian Law Reform Commission 2017). The ALRC, citing the Rochdale Borough Safeguarding Adults Board,^1^ point out that:

Some taxonomies of abuse also include “institutional abuse” as a form of abuse—described as occurring when the “routines, systems and regimes of an institution result in poor or inadequate standards of care and poor practice which affects the whole setting and denies, restricts or curtails the dignity, privacy, choice, independence or fulfilment of individuals.” (Australian Law Reform Commission 2017, 110)

## Results

### Sample of RACF

#### Demographics

Of the eighty residents whose records were reviewed, fifty-seven were female and twenty-three were male. Dementia was diagnosed in 70 per cent (95 per cent CI 55 to 81 per cent, 56/80) of residents. The mean age at death was eighty-nine years (SD 6.2; median 88.5; range 70–99 years). The mean age at death for residents with a PU, eighty-nine years (SD 5.8; median 89; range 78–99 years), was similar to the mean age at death for all residents without a PU, eighty-eight years (SD 6.5; median 88; range 70–99 years).

Of the eighty residents’ records reviewed, the source of admission for four residents was unknown. Of the remaining seventy-six, 58 per cent had been admitted from hospital, 30 per cent from home and 12 per cent from another RACF (table 1).

**Table 1:** Source of admission and presence of pressure ulcers

	% (95% Confidence Interval)	Old PU on admission % (95% Confidence Interval)	New PU post admission % (95% Confidence Interval)	Prevalence of PU in the last week of life
**Source of Admission**				
hospital	58 (41-73) [44]	17 (9-32) [7]	15 (8-28) [6]	15 [6]
home	30 (20-42) [23]	9 (2-33) [2]	35 (22-51) [8]	35 [8]
another RACF	12 (4-29) [9]	0 [0]	11 (2-45) [1]	11 [1]
unknown	[4]	[4]	[4]	[4]
**Total**	[80]	[13]	[19]	[19]

Of the forty-four residents admitted from hospital, 39 per cent (95 per cent CI 31 to 47 per cent, 17/44) had been hospitalized following a fall. Of these seventeen residents, 29 per cent (95 per cent CI 6 to 71 per cent, 5/17) had a fractured lower limb and 23 per cent (95 per cent CI 6 to 59 per cent, 4/17) had a fractured upper limb, including one concomitant fractured arm and leg.

The mean number of days to death, regardless of admission pattern, was 864 (SD 765; range 38–3459 days; median 584; LQ 328, UQ 1246). The mean number of days to death for residents admitted to a RACF from hospital was 694 days (SD 578; range 56–2219 days; median 540, LQ 257, UQ 860).

#### Prevalence of Pressure Ulcers on Admission and During the Last Week of Life

On admission to a RACF from hospital, four of forty-four residents developed a PU but were excluded from this analysis because the time of PU development was unknown. Of the remaining forty, 17 per cent were admitted with an old PU that had developed in hospital. Of the twenty-three residents admitted to a RACF from home, 9 per cent had an old PU on admission. Of the nine residents transferred from another RACF, none had a PU on admission to the study RACF (table 1).

There were twenty-seven residents who had at least one PU recorded in the last week of life, fifteen of whom developed a PU post admission, while nine residents were admitted to a RACF with a PU (including one resident who had both a PU on admission and developed one post admission).

#### Residents Identified on Admission as Being at Risk and Repositioned

Staff judged the majority (91 per cent) of residents to be at risk of PU on admission. Nearly all at-risk residents (96 per cent) had two-hourly repositioning, 24/7, documented in their records (table 2).

**Table 2:** Pressure ulcer risk and interventions

	% (95% Confidence Interval) [N = 80]
**MEDICAL AND NURSING RECORDS**	
**Diagnosis of dementia**	70 (55-81) [56]
**Behaviours of concern**	
Dementia	82 (64-92) [46]
**Type of restraint**	
Chemical	26 (12-35) [17]
Physical	1 ( 3-13) [5]
Both	5 (1-16) [4]
**Preventive interventions**	
Repositioned two-hourly	96 (88-99) [70]
Alternating pressure air mattress	5 (1-10) [4]
**Retrospective classification of at-risk**	
Classification of at-risk residents: immobility, residents requiring two staff assistance to walk, bedfast /wheel chair bound, two-hourly repositioning.	91 (75-97) [73]

#### Incidence of Pressure Ulcers After Admission in the Last Week of Life

After excluding residents with an unknown timing to PU development, the overall incidence of PU in the remaining sixty-seven at-risk residents who were repositioned was 21 per cent (95 per cent CI 13 to 31 per cent, 14/67). There was a significant difference (p = 0.04) in the risk of developing a new PU by the three admission sources. Four of forty-four residents admitted from hospital were excluded due to unknown timing of PU development. A total of fifteen residents developed a PU after admission from hospital, home, and/or a RACF. Of the forty admissions from hospitals with information on timing of a new PU, 15 per cent of residents developed a PU after admission that was present in the last week of life. Of the residents admitted from home, 35 per cent developed a PU after admission, and of the residents admitted from another RACF, 11 per cent developed a PU after admission that was present in the last week of life (table 1). Of the three residents judged to be at risk of a PU but not repositioned, none had a PU in the last week of life.

Of the fifteen residents who developed a PU after admission to a RACF that was present in the last week of life, 80 per cent of PUs were located on the sacrum (95 per cent CI 36 to 97 per cent, 12/15) and of these, 9/12 were stage 2 PUs. Of the 44 per cent (95 per cent CI 13 to 80 per cent, 12/27) of PUs on heels most (75 per cent, 9/12) were stage 2. Five residents developed PUs on both the sacrum and heels. Four (5 per cent) residents in our survey were provided with an APAM, of whom three were considered at risk of a PU. The timing of the provision of APAM in the nursing notes was ambiguous.

A death certificate was included in 15 per cent (12/80) of residents’ records and PU was noted on one (1/12) death certificate that did not attribute the PU as a contributory cause of death.

#### Behaviours of Concern and Restraints in Residents With and Without Dementia

Behaviours of concern were noted in 72 per cent of records for the last week of life. Of the residents with behaviours of concern, 15 per cent had a PU and all nine had been restrained in the last week of life, 22 per cent had been restrained but did not have a PU in the last week of life, 22 per cent were not restrained but had a PU, and 40 per cent were not restrained and did not have a PU. It is not known how often restraints were released during any shift or whether residents were restrained 24/7.

The prevalence of behaviours of concern displayed by residents with dementia was significantly greater (82 per cent; p = 0.028) than by residents without dementia (table 2). An additional two residents had been restrained without documentation of behaviours of concern. Documentation describing behaviours of concern included (verbatim):

- *becomes aggressive at night when woken to be repositioned*
- *hitting and kicking staff [and] refused care*
- *resistant to care*
- *very resistant when staff giving pressure care*
- *pushing staff hands away*
- *wincing when repositioned*
- *screams when woken to be repositioned*
- *yelling and screaming for help*

During the last week of life, residents who were repositioned two-hourly 24/7, and most likely for some months prior, were reported as uncooperative:

- *very sleepy this am [morning]*
- *too tired to join in activities*
- *won’t walk with physio[therapist]*
- *refusing food*
- *asleep at the table*

### Legal and Policy Material

#### Patients’ Rights

Australia and New Zealand recognize patients’ rights. In Australia, those rights are enshrined in the *Australian Charter of Healthcare Rights*^2^ (Australian Commission on Safety and Quality in Healthcare, 2008). For aged care, the *Charter of Care Recipients’ Rights and Responsibilities: Residential Care 2014* also outlines recipients’ rights. A key difference between the Australian Charter and the New Zealand *Code of Health and Disability Consumers’ Rights* is enforceability. The New Zealand version provides a codified benchmark against which the health complaints entity assesses complaints for potential breaches (Morris, Moore, and Bismark 2018, 219). By contrast, the Australian Charter “is in effect a guide only, is not specifically enforceable, and is not linked to the powers of the health complaints entities” (Morris, Moore, and Bismark 2018, 219). However, Australian patients may complain to the Health Complaints Commissions in the states and territories and/or the Aged Care Commissioner.

#### Regulation of Aged Care Facilities

In Australia, RACFs are regulated by state and Commonwealth legislation. The main Commonwealth statute is the *Aged Care Act 1997* (Cth). This Act concerns funding, allocation, licencing of approved providers, rights, and responsibilities. Importantly, it outlines the responsibilities of providers for the quality of care they provide and residents’ rights. When assessing the quality of care, the *Quality of Care Principles 2014* (Cth) may also be used as a benchmark.

An example of how the *Aged Care Act 1997* may be used to protect the elderly by regulating their RACF service providers is illustrated in a recent Australian Administrative Appeals Tribunal case. In *Saitta Pty Ltd v Secretary Department of Health and Aging* (2008) 105 ALD 55, the Administrative Appeals Tribunal found that:

A resident was observed an hour after breakfast … with a lap belt restraining her. It was Dr Lett’s oral evidence that, as well as restraining the resident from undertaking normal activity, this is also a dangerous practice as the resident may try to wiggle out causing skin to tear or bruise or the resident to fall and injure him/herself. This is an example of an incident where even if it was a once off occurrence the fact of it happening is strongly indicative of serious non-compliance, as the restraint could easily be removed when the resident had finished her meal. The Tribunal is satisfied that there is non-compliance with Standard Pt 3 Item 3.5 (residents to be assisted to achieve maximum independence), Item 3.6 (right to dignity) and Standard Part Item 4.4 (management actively working to provide a safe and comfortable environment consistent with residents’ needs).

The applicant’s approval as a provider of aged care services was revoked for several reasons, such as poor wound management and poor infection control. This case may set a precedent for future cases involving PUs, wound management, and the use of restraints.

The applicable legislation, the *Aged Care Act 1997*, does not prohibit the use of restrictive practices to manage the “behaviours of concern” of some residents in RACFs. However, there are protections available in civil law and international law. In civil law, the unlawful restraint of a person may constitute the tort of false imprisonment (*Zanker v Vartzokas* [1988] 34 A Crim R 11; *Bird v Jones* [1845] 7 QB 742). If the unlawful restraint is achieved by physical force, this may constitute the tort of battery (*Rixon v Star City* [2001] NSWCA 265). In international law, recent cases in the European Court of Human Rights, in relation to the use of restraints for adults with impaired capacity, illustrate a growing awareness of the importance of monitoring restrictive practices in aged care (*Re MLI* [2006] QGAAT 31; *Re WMC* [2005] QGAAT 26). In addition, restraints should only be used for the protection of the patient and not the convenience of staff (Staunton and Chiarella 2013). Carers who use excessive force and violence to restrain elders may be professionally disciplined (*Tasmanian Board of the Nursing and Midwifery Board of Australia v Wiggins* [2011] TASHPT 5). Despite the available legal options, the Australian Law Reform Commission (2017) has argued that reform is required to ensure that the *Aged Care Act 1997* (Cth) provides appropriate safeguards against elder abuse.

#### Torts and Crimes

For the defence of consent to be successful in either tort or crimes, there must have been a voluntary decision by a patient to allow a health practitioner to touch him/her in a specific way (*Re T (Adult): Refusal of Treatment* [1993] Fam 95; *Ljubic v Armellin* [2009] ACTSC 21). Accordingly, it may be possible for the courts and tribunals to find that repositioning constitutes unlawful touching if the resident did not consent to such touching.

The tort of negligence is also relevant to the repositioning of elders because health practitioners must maintain appropriate standards of care. In Australia, both the common law and legislation (Civil Liability Acts) define the parameters of the tort of negligence. The Australian Nursing and Midwifery Accreditation Council argued that repositioning residents may not breach the tortious standard of care (Australian Nursing and Midwifery Accreditation Council 2006). Despite the council’s claim, it remains open for the courts and tribunals to hold that such practices constitute a breach of the standard of care (s 5O, *Civil Liability Act 2002* (NSW); *Dobler v Halverson* [2007] NSWCA 335).

#### Coronial Jurisdiction

In Australia, coroners may investigate deaths that are considered “reportable” at law to determine the deceased’s identity and when, where, how, and why the deceased died (s6, *Coroners Act 2009* (NSW)). Coroners may investigate deaths that are attributable to PUs (*In the matter of an inquest into the death of Patricia Northcote* [2016] NSWCorC 2014/00034477 (5 April 2016); *In the matter of an inquiry into the death of Ms C* [2015] QLDCorC 2012/4591 (11 June 2015); *In the matter of an inquest into the death of Cynthia Thoresen* [2013] 2009/3 (22 May 2013); *In the matter of an inquest into the death of Maria Carmel Niceforo* [2016] WACorC 202/2014 (22 November 2016)). In addition, it is possible for coroners to use their statutory recommendation-making powers to direct suggestions about the prevention of PUs to RACFs (s3(e), *Coroners Act 2009* (NSW)). Coroners’ recommendations are intended to prevent future deaths and therefore are essentially public health and safety interventions (Bugeja, Woolford, and Willoughby 2017; Moore 2016).

## Discussion

We focused our examination of eighty records from eight RACFs on the last week of life for this labour intensive retrospective cross-sectional survey. Validation of our prevalence of PUs during the last week of life could not be performed using death certificates because documentation of PUs by medical staff, on the death certificates, was poor. Similar poor documentation on death certificates was noted in the United Kingdom (Cutting and White 2015).

There are 379 RACFs across the five Sydney local health districts (LHDs) (New South Wales Health Department 2017). The selection of the eight RACFs from two LHDs may limit the generalizability if our participating RACFs had lower rates of PUs than non-participating RACFs. We believe the results from our eight RACFs can be generalized to the remaining fifty-seven RACFs within the same LHD, as each RACF was randomly selected and represents eight different postal codes within the two LHDs.

There is a temporal limitation in prevalence surveys. The temporal limitation in our study is the missing documentation of the period of immobility from the notes. Although causality between the period of immobility for our fifteen residents who developed a new PU after admission cannot be determined, it is accepted that damage to skin and deeper tissue occurs during periods of immobility as short as half an hour (Bliss 1994) and up to four to six hours (Salcido 2004; Linder-Ganz and Gefen 2004; Gefen 2008; Exton-Smith, Overstall, and Wedgewood 1982). Regardless of the limitation to the design of all prevalence surveys, the measure of prevalence emphasizes the burden of painful PUs in 70 per cent, the majority, of our residents. The incidence indicates the success of preventive practices during residency at the study RACFs. However, 21 per cent of residents developed a new PU suggesting that routine two-hourly repositioning failed.

A final possible limitation of this survey was a poor temporal relationship between the development of each PU and the timing of restraints. Regardless of timing before or after PU development, restraints prevent voluntary repositioning, and unrelenting pressure will result in discomfort and persistent pain (Woo et al. 2017; Gorecki et al. 2009; Bliss 2009).

Admissions from hospital accounted for half of our residents, and these residents were more likely to have a PU than residents admitted from the community or from other RACFs. Over a third of our RACF residents admitted from hospital had suffered a fall and fractures that would have resulted in periods of immobility, placing them at risk of PU. Three decades ago, a study revealed that sixty patients with hip fractures in the United Kingdom developed 124 PUs, mainly on the sacrum (45 per cent) and heels (23 per cent), supporting our concerns that immobility due to fractures may be a proxy risk for PU (Versluysen 1985). Residents with a fracture need relief of pressure. However, residents with a fracture may suffer pain and distress during manual repositioning. This can be avoided with an APAM while preventing a PU.

In 635 patients with hip fractures in six European countries, 10 per cent had a PU on arrival to hospital, while twice as many, 22 per cent, had a PU on discharge from hospital (Lindholm et al. 2008). Most commonly, PUs develop on the sacrum and heels in bed-bound residents (Rich, Margolis, and Shardell 2011; Exton-Smith and Sherwin 1961) and the ischial tuberosities in wheelchair-bound residents (Chaves et al. 2007; Anthony, Barnes, and Unsworth 1998).

Repositioning is currently the method of PU prevention globally as well as in Australia (National Pressure Ulcer Advisory Panel, European Pressure Ulcer Advisory Panel, and Pan Pacific Pressure Injury Alliance 2014). Therefore, our observation that PUs remain highly prevalent suggests that two-hourly repositioning has not sufficiently impacted this prevalence. Bearing in mind that wide variation exists in the prevalence and incidence rates of PUs in the elderly, 34 per cent of residents with PUs in our study is in line with global findings of a wide variety of staging in PU from 9 per cent (Moore and Cowman 2012), 10 per cent (Clinical Excellence Commission 2017), 15 per cent (Wilborn, Halfens, and Dassen 2006), 26 per cent (Keelaghan et al. 2008), 37 per cent and 53 per cent (Davis and Caseby 2001). The variation in the rates may be due to differences in the population of patients studied, data collection, study methodology, and care provided (Tannen, Dassen, and Halfens 2009; Baharestani et al. 2009). Repositioning two-hourly 24/7 did not prevent PUs in many of our residents. Pressure relief should be provided in the form of an APAM, not waking residents up for the purpose of repositioning. An APAM provides pressure relief to all parts of the body every few minutes throughout the twenty-four hours without waking residents, whereas repositioning for pressure relief is usually only carried out two-hourly. It is unacceptable that this prevalence of PUs be allowed to continue.

In the last week of life, nearly all (91 per cent) residents were judged to be at risk of a PU and nearly all of those at risk (96 per cent), were repositioned two-hourly 24/7. Yet, one third of residents who were repositioned 24/7 had one or more PUs at the time of death, raising the question that a PU risk screening tool in clinical practice is not protecting vulnerable residents (Anthony et al. 2010; Kottner and Balzer 2010). The speed of screening is imperative, and following the “golden hour” rule for risk of PU encourages rapid screening (Sharp and White 2015) by a multidisciplinary team (Kayser-Jones, Beard, and Sharpp 2009; Sharp and White 2015; Bliss 2005) who then provide at-risk residents with an APAM (Exton-Smith, Overstall, and Wedgewood 1982; Bliss, McLaren, and Exton-Smith 1967) within an hour (Gefen, Farid, and Shaywitz 2013; Stekelenburg et al. 2008). This rapid intervention reduces the likelihood of a new PU and disruption to sleep.

### Neglect or Abuse

It is difficult to determine whether the development of PUs is neglect and/or abuse when nurses and care staff in RACFs lack the authority to procure pressure-relieving equipment such as APAMs. Pressure ulcers can start to develop in four to six hours (Salcido 2004, Linder-Ganz and Gefen 2004; Gefen 2008; Exton-Smith, Overstall, and Wedgewood 1982) or even as little as half an hour according to Bliss (1994), yet access to pressure relieving equipment can take up to two days (Sharp et al. 2000) or may never be accessed. Consistent with public health principles, we believe that a better approach is the use of the available legal tools. For example, section 3(item 3.2) of the Australian *Quality of Care Principles 2014* (*Cth*) of the *Quality of Care Principles (Cth)* provides care staff with an option that may prevent PUs: “… an air mattress appropriate to each care recipient’s condition.”

Regardless of dementia, the use of two-hourly repositioning is harmful, and residents with dementia have no ability to provide informed consent.

We concur with others who have shown that the ritualistic practice of waking residents every two hours for the purpose of repositioning contributes to severe sleep deprivation and behaviours of concern (Cohen-Mansfield and Marx 2016). Sleep is a fundamental phenomenon in most organisms and the sleep–wake cycle is a physiological rhythm which modulates endogenous neuronal activity in the brain (Roh and Holtzman 2015). Similar to smoking or drug use, the immediately visible physical impact does not reflect the dysfunction caused in brain mechanisms due to sleep deprivation. Chronic sleep deprivation can cause significant and cumulative physiological deficits and the disruption of normal neurophysiological mechanisms (Chittora et al. 2015; Seyffert and Berofsky-Seyffert 2015). However, in clinical practice, it is often difficult to distinguish pain-related behaviour from behavioural symptoms related to other disorders, such as anxiety disorder, or to dementia-related behaviours (van Dalen-Kok et al. 2018).

We also believe that this clinical practice of two-hourly repositioning may breach the *Optional Protocol to the Convention Against Torture 2002* (OPCAT). In some cases, the OPCAT may provide a mechanism for complaints about elder abuse. The OPCAT promotes independent, regular visits by international and national bodies to monitor conditions within settings where people are deprived of their liberty (Weller 2017, 44). The OPCAT requires states to establish a national system of inspections of all places of detention, and this could include RACFs (Australian Law Reform Commission 2014, 247).

Because Australia is a dualist jurisdiction, international documents must normally be incorporated into domestic legislation. Australia has not yet ratified the OPCAT (Australian Law Reform Commission 2017, 155). However, according to a growing number of commentators, ratification may not be necessary (Simma and Alston 1992; Weller 2017). Commentators have recently emphasized that there is arguably a “common law of human rights” which jettisons the need for formal ratification (Simma and Alston 1992; Weller 2017, 20).

We have described examples of “triggers” for behaviours of concern possibly caused by sleep disruption. The prevalence of behaviours of concern displayed by our residents with dementia was significantly greater than by residents without dementia. Just a few years ago, 53 per cent of all residents in Australian RACFs suffered from dementia (Australian Institute of Health and Welfare 2011) and that was similar to the prevalence of dementia (54 per cent) in a Belgian RACF study (Vandervoort et al. 2013). But the majority (70 per cent) of our residents suffered from dementia. Adults with dementia may forget how to move (Reisberg 1984), and the neuro-pathology of dementia is thought to disrupt sleep (Vitiello and Borson 2001). Poor night-time sleep, regardless of dementia, results in excessive daytime sleepiness (Weinhouse et al. 2009, Cole and Richards 2007). With the majority, 73/80, of all residents having been repositioned two-hourly 24/7 we believe the genesis of behaviours of concern is likely to be severe sleep deprivation.

There is no empirical evidence that anyone with dementia experiences less pain, and therefore behaviours of concern may also have a genesis of pain. Cognitively impaired elderly persons whose verbal fluency has declined have been identified as having an altered expression of pain (Horgas, Elliott, and Marsiske 2009), making the location of pain or cause of the pain difficult for staff to identify (Manfredi et al. 2003).

Restraint authorizations were documented for twenty-two residents exhibiting behaviours of concern, yet pain due to PU development (Dallam et al. 1995; Pieper, Langemo, and Cuddigan 2009; Ahn, Stechmiller, and Horgas 2013) can result in residents screaming. If residents are unable to inform staff that they are in pain, the screaming may be interpreted by staff as behaviours of concern. Restraints used on one-third of our residents may have been responsible for 15 per cent developing a PU or responsible for unrelenting pain, with residents with late dementia being unable to express pressure pain and discomfort.

Currently there is a dearth of evidence for the prevention of PU. The international *Clinical Practice Guideline* (National Pressure Ulcer Advisory Panel, European Pressure Ulcer Advisory Panel, and Pan Pacific Pressure Injury Alliance 2014) includes only seventy-seven evidence-based statements. The remaining 498 statements, including repositioning frequency, are based on expert opinion only (Black 2015). Two-hourly repositioning 24/7 has not resulted in the elimination of PUs (Krapfl and Gray 2008; Clark 1998, Gillespie et al. 2014, Rich, Margolis, and Shardell 2011) but care staff are required to follow the RACF care plans and reposition residents two-hourly. This practice continues despite the adverse effects to residents we have documented. We believe the practice of 24/7 two-hourly repositioning may be unintentional institutional abuse of elders.

The World Health Organization has estimated that the prevalence rate of elder abuse in high- and middle-income countries ranges from 2 to 14 per cent (Australian Law Reform Commission 2016, 11). We believe these figures are an underestimation for several reasons. First, the abused person may not want the abuser to be investigated or prosecuted (Australian Law Reform Commission 2017, 392). Second, elder abuse tends to be invisible (New South Wales Parliament 2016). Third, two-hourly repositioning 24/7 is carried out to prevent PUs, but it is not currently recognized as a form of unintentional institutional elder abuse.

### Potential Legal Implications

The rise in litigation against RACFs in Australia (Nelson 2003), the United Kingdom (Toolan et al. 2014), and the United States (Stevenson and Studdert 2003) suggests RACF service providers who fail to meet standards of care may be legally liable. As outlined in the results section above, there are numerous legal options (such as patients’ rights) to improve the quality and safety of care for the elderly in RACFs and prevent PUs.

### Patients’ Rights

In addition to the domestic *Charter of Rights* that we discussed in the results section above, there is international law which recognizes patients’ rights. The United Nations International Plan of Action adopted by all countries in April 2002 recognizes the importance of elder abuse and includes it in the framework of the *Universal Declaration of Human Rights* (*UDHR*). Preventing elder abuse in an ageing world is the responsibility of all who interact with the elderly (World Health Organization 2002). Pursuant to article 5 of the *UDHR*, and article 7 of the International Covenant on Civil and Political Rights, “no one shall be subjected to torture or to cruel, inhuman or degrading treatment or punishment.” These international instruments may also be used to advance efforts to protect our elders because they may be applied to the unlawful use of restraints and unlawful repositioning.

Competent patients have a legal right to refuse treatment (*Re PVM* [2000] QGAAT; *Schloendorf v Society of New York Hospital* 195 NE 92 [1914]). Consent is both a defence to claims of wrongful touching of the body and a negligence-based duty to provide information to patients (Stewart 2017). Repositioning elderly residents may be unlawful if residents do not consent to repositioning and/or if they refuse to be repositioned. In *Dean v Phung*,^3^ Basten JA set out how consent would be given in this context: consent is validly given for medical treatments where the patient has been given basic information about the nature of the treatment. Despite the problems of obtaining informed consent, nurses use the following instructions to residents as consent, “We have to roll you over [reposition you] so you don’t get pressure ulcers.” Regardless of our residents objecting to being repositioned, nurses will continue to reposition residents as directed and in accordance with the RACF’s care plan. Regardless of a diagnosis of dementia or not, the use of two-hourly repositioning is harmful. For residents with dementia, the harm continues as there is no ability for them to make or withdraw consent. We did not search for any documentation referring to consultation with the enduring guardian. Even with an enduring guardian or “person responsible,” this practice may still constitute unintentional elder abuse. Sometimes, protective measures may conflict with a person’s autonomy, such as where an older person refuses to accept support. Where possible, the ALRC has sought to recommend changes to the law that both uphold autonomy and provide protection from harm, but where this is not possible, greater weight is often given to the principle of autonomy (Australian Law Reform Commission 2017, 20).

The courts have attempted to establish principles. For example, in *Re MB (Medical Treatment)*^4^ the court considered that the inability to make a decision (incapacity) exists in the following circumstances:

- when a person is unable to comprehend and retain information which is material to a decision, especially the likely consequences of not having the treatment;
- where he or she is unable to use the information which is material to a decision, especially the likely consequences of not having the treatment;
- where he or she is unable to use the information and weigh it up as part of the balancing process in arriving at the decision.

### Coronial Jurisdiction

As we outlined in the results section above, the coronial jurisdiction is another legal avenue which has the potential to advance efforts to prevent PUs. Coronial jurisdiction commentators increasingly recognize the powerful preventive potential of coroners’ recommendations (Freckelton and Ranson 2017, 584; Moore 2016). Coronial information is of particular importance to policymakers, organizations, practitioners, and researchers who have an interest in mortality and morbidity prevention. Many organizations analyse coroners’ recommendations for patterns, so that these trends can inform and improve their work. Autopsies are rarely performed on RACF residents with PUs. Visual inspection suffices the diagnosis of a PU, and we are not suggesting PU is usually the cause of death, rather residents died with a PU. Consequently, death certificates rarely include mention of a PU or state that PU is a cause of death. Coroners’ recommendations about PUs could be used by RACFs as a prevention tool. Therefore, we argue that coroners’ recommendations have the potential to prevent PUs and deaths attributable to PUs. Unfortunately, in relation to RACF deaths in Australia, coroners’ recommendations were made in less than 2 per cent for external cause of deaths (Bugeja, Woolford, and Willoughby 2017). The paucity of such recommendations in deaths in RACFs highlights potentially missed opportunities for the identification and promotion of injury prevention interventions (Bugeja, Woolford, and Willoughby 2017).

## Recommendations

We propose seven major changes in an effort to reduce harm and unintended abuse of the elderly:

- Cease 24/7 repositioning.
- Provide every RACF bed an APAM, or at the very least provide an APAM to residents identified at-risk of PU without delay.
- Cease physical restraints.
- Introduce a Convention on the Rights of Elders.
- Clarify the legal definition of institutional elder abuse for RACFs and include this definition in national legislation.
- Amend the Coroner’s Court legislation in all Australian jurisdictions to provide coroners with a mandatory, statutory authority to investigate deaths from PUs in RACFs.
- Provide training for RACF service providers, registered nurses, and care staff on health and human rights (pursuant to General Comment 14, para 44 (2000) as adopted by the Committee on Economic Social and Cultural Rights).

The *Clinical Practice Guideline* (National Pressure Ulcer Advisory Panel, European Pressure Ulcer Advisory Panel, and Pan Pacific Pressure Injury Alliance 2014) must be amended to ensure risk screening is carried out within a “golden hour” (Sharp and White 2015) and an APAM provided immediately. Optimal time for screening for risk is not a replacement for two-hourly repositioning as a preventive intervention. A rapid and accurate identification of at-risk residents for PUs would enable an immediate intervention, such as use of an APAM.

A new Convention on the Rights of Older People is essential in order to understand and disseminate the basic facts about preventing PUs. The elderly at risk of PUs suffer because even if policies are put in place for the provision of APAMs, these policies are rarely implemented. The majority of our residents were not placed on an APAM, so when PUs developed in a third of residents, their lives were imperilled and possibly shortened. Submissions to the ALRC Inquiry (2017) have already identified PUs as a problem (Australian Law Reform Commission 2017, 122).

To be effective, components of a new Convention on the Rights of Older People should include, but would not be limited to, APAMs on every bed in every RACF, training of all care providers and healthcare personnel, and access to healthcare workers with relevant training in geriatric, dementia, and palliative care

These recommendations will enable residents to sleep undisturbed all night. Being able to sleep may reduce or eliminate behaviours of concern and prevent daytime sleepiness. Exploration by staff to determine the exact nature of sleep problems of agitated residents may identify other sleep-waking practices that need to be discontinued.

## Conclusion

Within the RACF environment where 24-hour nursing care is provided, a lack of suitable equipment or staff are not acceptable excuses for PU development (Needleman et al. 2011). We believe two-hourly repositioning could be unintentional institutional abuse rather than a preventive safety practice. It is time to protect our elderly in RACFs in the last years of their lives. On occasions it may be necessary to physically restrain residents for safety if they pose a threat to themselves or others (Kerridge, Lowe, and Stewart 2013) but for short periods only and with an alternating pressure air cushion or APAM. If medication is required to settle residents, it must be for short periods only and with an alternating pressure air cushion or APAM if the medication causes immobility.

We cannot prevent PUs without acknowledging that PUs still occur in a third of our most frail residents at the end of their lives. Two-hourly repositioning is a poor preventive practice of PUs (Hagisawa and Ferguson-Pell 2008; Krapfl and Gray 2008; Clark 1998; Rich, Margolis, and Shardell 2011; Defloor, De Bacquer, and Grypdonck 2005; Gillespie et al. 2014). Repositioning results in severe sleep deprivation, and restraints may be unlawful. Unlawful repositioning and restraining of residents is elder abuse and should cease immediately.

Most RACF service providers strive to provide lawful and ethical quality of care for all residents. However, the evidence points away from this conclusion in relation to PU prevention.

Service providers in RACFs need to better recognize harm to residents, manage legal risks, and enhance the quality and safety of care provided to institutionalized elders.

## Footnotes

1 Rochdale Borough Safeguarding Adults Board, Institutional Abuse <www.rbsab.org>

2 Australian Charter of Healthcare Rights https://www.safetyandquality.gov.au/national-priorities/charter-of-healthcare-rights/.

3 [2012] NSWCA 223

4 Re *MB (Medical Treatment)* [1997] 2 FLR 426 at 224.
